# Brain Activity in Advantageous and Disadvantageous Situations: Implications for Reward/Punishment Sensitivity in Different Situations

**DOI:** 10.1371/journal.pone.0080232

**Published:** 2013-11-12

**Authors:** Guangheng Dong, Xiao Lin, Yanbo Hu, Qilin Lu

**Affiliations:** 1 Department of Psychology, Zhejiang Normal University, Jinhua, Zhejiang Province, P.R. China; 2 Center for Integrative Neuroscience and Neurodynamics (CINN), School of Psychology and Clinical Language Sciences, University of Reading, United Kingdom; 3 School of Life Science, University of Science and Technology of China, Hefei, Anhui Province, PR; University Of Cambridge, United Kingdom

## Abstract

**Objective:**

This study modeled win and lose trials in a simple gambling task to examine the effect of entire win–lose situations (WIN, LOSS, or TIE) on single win/lose trials and related neural underpinnings.

**Methods:**

The behavior responses and brain activities of 17 participants were recorded by an MRI scanner while they performed a gambling task. Different conditions were compared to determine the effect of the task on the behavior and brain activity of the participants. Correlations between brain activity and behavior were calculated to support the imaging results.

**Results:**

In win trials, LOSS caused less intense posterior cingulate activity than TIE. In lose trials, LOSS caused more intense activity in the right superior temporal gyrus, bilateral superior frontal gyrus, bilateral anterior cingulate, bilateral insula cortex, and left orbitofrontal cortex than WIN and TIE.

**Conclusions:**

The experiences of the participants in win trials showed great similarity among different win–lose situations. However, the brain activity and behavior responses of the participants in lose trials indicated that they experienced stronger negative emotion in LOSS. The participants also showed an increased desire to win in LOSS than in WIN or TIE conditions.

## Introduction

A reward/punishment is an attractive/disgusting stimulus given to an individual to alter his or her behavior. People's experience of the same stimulus may be altered by their specific situation (i.e., an advantageous or disadvantageous situation). Many idioms and phrases describe this phenomenon. For example, “another person helped me” may be expressed as “another person gave me timely assistance” in a disadvantageous situation or “another person made perfection even more perfect” in an advantageous situation. Despite much interest in the valuation of reward/punishment in different situations, the neurobiological basis of how the valuation process works have received little research attention.

This study assessed brain activity in different win–lose situations and related reward/punishment experiences. To create different win–lose situations, we designed a guessing task with a purported win/loss rate of 50% for each trial. Although participants were told that the outcomes were random, the outcomes were predetermined and classified into three win–lose situations: the participants double their initial balance (WIN), they lose all they have (LOSS), and win/loss no more than 10 yuan (TIE). These WIN or LOSS series were included to create different win–lose situations. The TIE was used as a baseline.

Brain imaging studies on reward/punishment have revealed several related brain regions during this mental process. First, the reward system is a collection of brain structures, which include the ventral tegmental area, the ventral striatum, and a part of the prefrontal cortex; these structures regulate and control behavior by pleasurable effects [1], [2]. The orbitofrontal cortex (OFC) is also important in signaling expected rewards/punishments and is activated by reward [3]–[5].

The insula significantly affects the experience of emotions [6], [7]. A large volume of anterior insula gray matter correlates with increased accuracy in subjective sense of negative emotional experience [8]. The disadvantageous situation (LOSS) elicits negative emotion [6], [7] and affects the experience of subsequent reward/punishment. These effects are indicated by activity in the insula. Thus, we hypothesized that LOSS elicits more intense insula activity than WIN and TIE.

Second, emotion affects the experience of expected reward/punishment. Several brain regions, such as the orbitofrontal gyrus, ventral striatum, and prefrontal gyrus [3], [4], [9], are responsible for the reward/punishment experience. We hypothesized that disadvantageous conditions elicit more intense brain activity in these related brain regions because they elicit stronger emotion than other conditions.

Third, during the reward/punishment experience, individuals regulate their emotions (whether negative or positive) and thus activate brain regions related to executive control, such as the anterior cingulate cortex (ACC) [10]–[12], and areas that regulate emotion (i.e., the dorsomedial and ventrolateral prefrontal cortex). The LOSS situation elicited strong negative emotion and thus required greater executive control during this process. Thus, we hypothesized that LOSS increases effort in regulating emotional experience, as indicated by intense activity in related brain regions.

## Methods

### Participant Selection

‘The Human Investigations Committee at Zhejiang Normal University’ approved this study (zjnuhe09062). All participants provided written informed consent. Seventeen healthy young adults (age: 21.3±1.7 years; female: 5) participated in this study. None of them reported current Axis I disorders as assessed using structured psychiatric interviews (M.I.N.I.) [13] by an experienced psychiatrist. Depression was assessed using the Beck Depression Inventory [14] with an exclusionary cut-off of >5. All subjects are right handed and have not suffered any head injury with loss of consciousness during their lifetime.

### Tasks and Procedure

**Figure 1:**
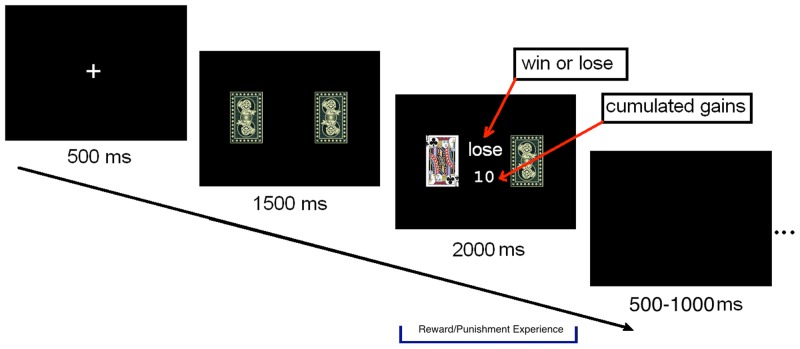
The timeline of one trial in present task. First, the backsides of two playing cards were shown and participants were asked to choose either the right or the left card with a button press. After 1.5(red playing cards, including the heart and diamond J, Q, K) or lost (black playing cards; including the spade and club J, Q, K) 10 Yuan.

A reality-simulated guessing task was designed to create win or loss context [15]. Figure 1 shows the event sequence of each trial during the task. A white cross would be presented at the center of a black screen for 500 ms to cue the beginning of a new trial. Then the backs of two cards were shown side by side and participants were asked to choose either the right or the left one with a button press as fast as possible. The selected card would be turned over after it was presented for 1500 ms and displayed for another 2000 ms. Participants would win 10 Chinese Yuan (≈$1.5) if the selected card was red or lose 10 Chinese Yuan if it was black. The word “win” or “loss” appeared between the two cards for 2000 ms immediately after the turn over of the selected card. The accumulated balance was presented beneath the word. The win or lose trials were presented randomly throughout the task. A black screen would be presented for 500 ∼1000 ms before the appearance of the next white cross [15]. The whole task consisted of 245 trials grouped into two blocks; one of them consisted of 120 trials while the other consisted of 125 trials with 60 s between blocks. E-prime software (Psychology Software Tools, Inc.) was used to present the task and acquire task-performance data while participants was in the scanner. After finishing the gambling task in the scanner, participants were asked to answer a 6-item questionnaire about their experiences in different win-lose situations out of scanner (Table S1).

**Figure 2:**
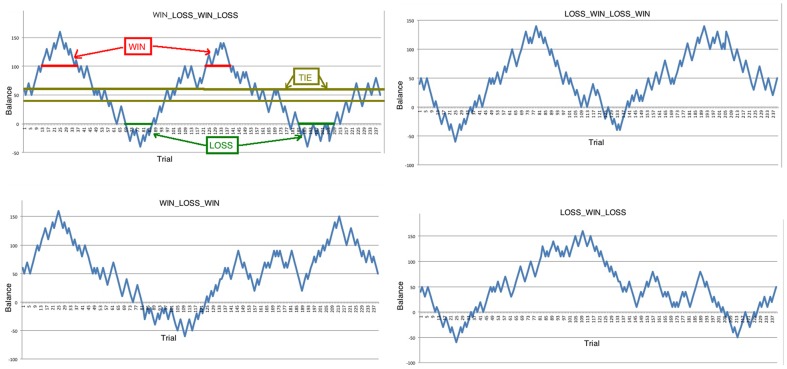
The figure shows the changes of the whole balance in this study. Participants' balance will waved in one of the four situations (WIN-LOSS-WIN-LOSS; WIN-LOSS-LOSS-WIN; LOSS-WIN-LOSS-WIN; LOSS-WIN-WIN-LOSS).

Each participant was provided 50 Yuan as the initial balance before the task, and was explicitly informed that he or she would receive the entire balance in cash at the end of the task. Although participants were told the outcomes are ‘random’, we designed three different conditions: (1) WIN, the balance is over 100 Yuan (participants doubled their initial balance). (2) LOSS, the balance is less than 0 Yuan (participant lost all they have); and (3) TIE: the balance is between 40 to 60 Yuan (participants win or lose no more than 10 Yuan). See Figure 2. Participants who choose the same card for more than 75 percent of all trials (they show button bias) or choose the same card for more than 10 continuous trials (they might not focusing on the task) were excluded from further analysis.

### Image Acquisition and Pre-processing

Structural images covering the whole brain were collected using a T1-weighted three-dimensional spoiled gradient-recalled sequence (176 slices, TR = 1700 ms, TE = 3.93 ms, slice thickness = 1.0 mm, skip = 0 mm, flip angle = 15°, inversion time 1100 ms, field of view = 240*240 mm, in-plane resolution = 256*256). Functional MRI was performed on a 3T scanner (Siemens Trio) with a gradient-echo EPI T2 sensitive pulse sequence in 33 slices (interleaved sequence, 3 mm thickness, TR = 2000 ms, flip angle 90°, field of view 220×220 mm^2^, matrix 64×64). Stimuli were presented using the Invivo synchronous system (Invivo Company, www.invivocorp.com/) through a screen in the head coil, enabling participants to view the stimuli.

### First-level Regression Analysis

Imaging analysis was conducted using SPM5 (http://www.fil.ion.ucl.ac.uk/spm). Images were slice-timed, reoriented (manual), and realigned to the first volume. T1-co-registered volumes were then normalized to an MNI T1template and spatially smoothed using a 6 mm FWHM Gaussian kernel. In this study, we only paid attention to the reward/punishment experience process. Every condition (WIN, LOSS, TIE) consisted of 40 valid trials for each participant.

A general linear model (GLM) was applied to identify blood oxygen level dependence (BOLD) activation in relation to separate event types. There were seven types of trials: win or lose trials in WIN, LOSS, and TIE conditions; besides this, the OTHER (trials of no interest) were also included in the design matrix but not analyzed in the future. The six head-movement parameters derived from the realignment stage were included as covariates of no interest. In addition, reward history (win or loss before the present trial (50, 40, 30, …)), and the response action (left (1) vs. right (2)) were also modulated as covariance to eliminate their potential influence to the results. These regressors were time-locked to the onset of the target and sustained the same duration. GLM was independently applied to each voxel to identify voxels that were significantly activated for the different events of each condition.

### Second-level Group Analysis

Second level analysis treated inter-subject variability as a random effect. First, we determined voxels showing a main effect in WIN, LOSS and TIE conditions relative to implicit baseline. Second, we tested for voxels that showed higher or lower activity in six contrasts of interest (lose (WIN-TIE, LOSS-TIE, WIN-LOSS); win (WIN-TIE, LOSS-TIE, WIN-LOSS)). We first identified clusters of contiguously significant voxels at an uncorrected threshold *p*<0.01, as also used for display purposes in the figures. We then tested these clusters for cluster-level FWE correction *p*<0.01 and the AlphaSim estimation indicated that clusters with 30 contiguous voxels would achieve an effective FWE threshold p<0.01 The smoothing kernel used during simulating false-positive (noise) maps using AlphaSim was 6.0 mm, and was estimated from the residual fields of the contrast maps being entered into the one-sample t-test. The formula used to compute the smoothness is that used in FSL (see http://www.fmrib.ox.ac.uk/analysis/techrep/tr00df1/tr00df1/node6.html for more information).

## Results

### Subjective experience reports

In win trials, all participants reported positive experiences no matter in disadvantageous situations (LOSS) [*M = 5.53, SD = 0.624*] or in advantageous situations (WIN) [*M = 5.71, SD = 0.470*], no significant difference were found between these two conditions [*t* = −1.144, *p* = 0.269].

In loss trials, participants reported more [*t* = −3.516,*p* = 0.003] negative emotion in LOSS [*M = 1.94, SD = 0.827*] than in WIN [*M* = 2.94, *SD* = 0.899]. Participants' craving for win in LOSS [*M = 6.59, SD = 0.507*] is significantly higher [*t* = 5.098,*p* = 0.001] than that in WIN [*M = 5.29, SD = 0.772]*.

### Correlation results

Beta value of the brain activation was used to perform the correlation analysis. The beta values were the peak values within a give cluster, and the values were detected by the software neuroelf (http://neuroelf.net/).

**Figure 3:**
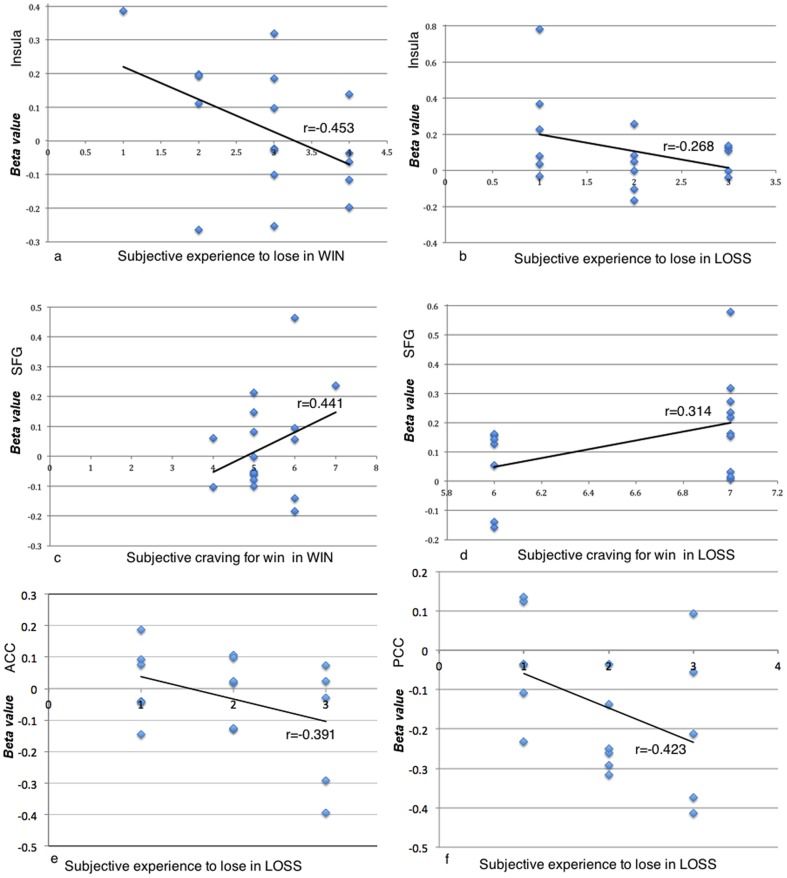
Correlations between brain activations and self report experience. **a**) Correlation between brain activations in insula and the participants' experiences to lose in WIN condition. **b**) Correlation between brain activations in insula and the participants' experiences to lose in LOSS condition. **c**) Correlation between brain activations in SFG and the participants' craving for win in WIN condition. **d**) Correlation between brain activations in SFG and the participants' craving for win in LOSS condition. **e**) Correlation between brain activations in ACC and the participants' experiences to lose in LOSS condition. **f**) Correlation between brain activations in PCC and the participants' experiences to lose in LOSS condition.

Our results showed that the activities in insula marginally significantly correlate with subjective rating of negative emotion in WIN condition (*r* = −0.453, *p* = 0.058) (Figure 3a). No significant correlation was observed between the activities in insula and LOSS condition, or between activities in SFG with WIN and LOSS conditions (*r* = 0.441, *p* = 0.076; *r* = 0.314, *p* = 0.220) (Figure 3c, 3d). Marginally significant correlations were found between ACC (*r* = −0.391, *p* = 0.084) and PCC (*r* = −0.423, *p* = 0.064) activation and subjects' experience to lose in LOSS (Figure 3e, 3f).

### Imaging Results

We compared the brain activities in win and lose trials in different comparisons (WIN-TIE, LOSS –TIE and WIN-LOSS).

### Reward/Punishment Sensitivities in Win Trials

**Figure 4:**
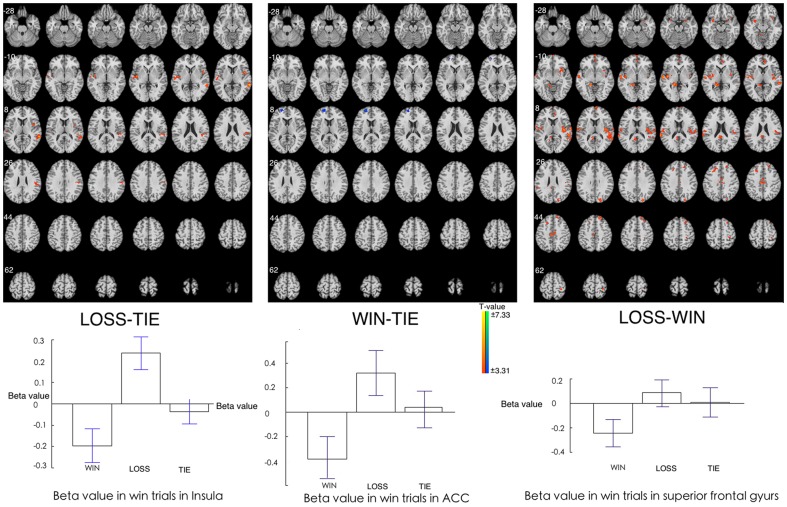
Brain areas showing different activations after win trials among WIN, LOSS, and TIE situations. **Upper**: Higher activation is found in posterior cingulate cortex in LOSS to TIE condition. No difference was found in other comparisons. **Bottom**: Beta figures in PCC in win trials in different situations.

Participants' responses to win trials were not differing much in different situations (WIN, LOSS, TIE). The only difference was found when comparing LOSS to TIE (lower brain activation in posterior cingulate). Besides this, no significant difference was found in other comparisons (WIN-TIE, LOSS-WIN). Beta figure of in the PCC showed the difference was caused by the decreased brain activation in LOSS (Figure 4, Table 1).

**Table 1:** Regional brain activity changes in different comparisons

	x,y,z a	Peak intensity (T-value)	Cluster size b	Region c	Brodmann's Area
**Difference in win trials**					
LOSS - TIE (Lower activated)	3, −57, 27	−4.785	32	R Posterior cingulate cortex	31
					
**Difference in lose trials**					
LOSS -TIE (Higher activated)	66, −42, 6	5.624	142	R Middle Temporal Gyrus	21
	−42, −9, −3	4.586	60	L Insula	
WIN -TIE (Lower activated)	−21, 54, 15	−4.016	46	L Superior Frontal Gyrus	8,9
LOSS -WIN (Higher activated)	−57, −15, 0	5.431	105	L Superior Temporal Gyrus	41,42
	−69, −39, 18	3.689	44	L Superior Temporal Gyrus	41
	63, −33, 9	7.134	282	R Superior Temporal Gyrus	41,42
	3, 69, −9	4.006	45	R Superior Frontal Gyrus	8,9
	15, 39, 48	5.015	59	R Superior Frontal Gyrus	8,9
	−27, 24, 39	3.587	38	L Orbitofrontal Cortex	11
	9, 39, 3	3.592	33	R Anterior Cingulate	32
	−9, −24, 42	4.283	55	L Posterior Cingulate	31
	33, 15, −9	5.620	42	R Insula	
	−42, −12, 0	4.736	60	L Insula	

a Peak MNI Coordinates.

b Number of voxels. *p*<0.01 FWE corrected and at least 30 voxels. Voxel size = 3*3*3.

c The brain regions were referenced to the software Xjview (http://www.alivelearn.net/xjview8) and double checked with atlas.

### Reward/Punishment Sensitivities in Lose Trials

Robust brain activations were found in loss trials among WIN, LOSS, and TIE. During the LOSS relative to TIE, the greater BOLD signals were observed in right middle temporal gyrus, and the left insula cortex. In WIN, relative to TIE, the lower brain activation was found in the left superior frontal gyrus (SFG). The most robust brain activities were found when comparing LOSS to WIN, higher brain activations were found in right superior temporal gyrus (STG), bilateral superior frontal gyrus, right ACC, bilateral insula cortex, and left orbitofrontal cortex.

**Figure 5:**
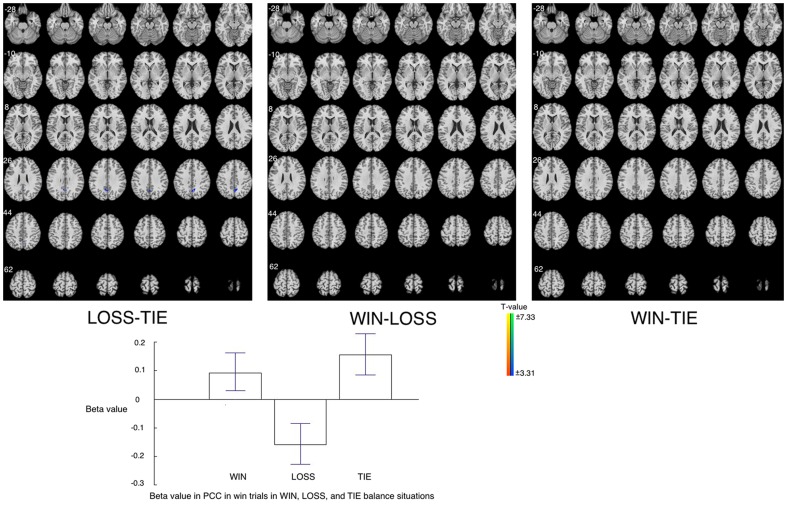
Brain areas showing different activations after lose trials among WIN, LOSS, and TIE situations. Upper: Comparisons among WIN, LOSS and TIE conditions after loss trials. Bottom: The Beta figures in Insula, ACC, and SFG in lose trials in different situations.

Beta figures showed that the differences in insula and SFG were caused by the enhanced brain activations in LOSS. In addition, the difference in ACC was caused by the decreased brain activation in WIN and increased brain activation in LOSS (Figure 5, Table 1).

### Insula activation in different balance situations

**Figure 6:**
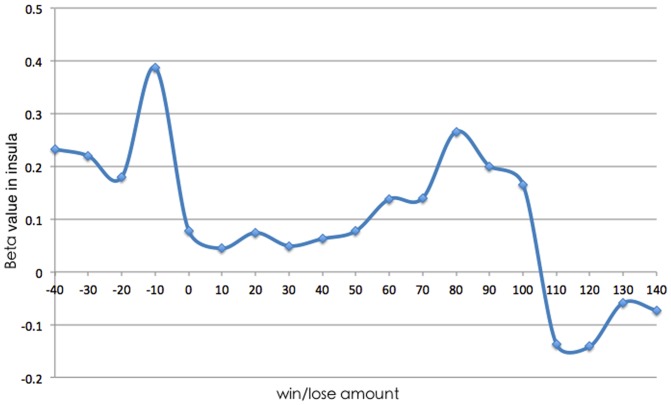
Beta values in insula in different win/lose situations.

To confirm that the separation of WIN and LOSS was reasonable, we selected the insula as the region of interest and measured its brain activity (peak beta value) at different amounts won/lost (−40 to 140). Figure 6 shows that brain activity significantly changed at 0–10 and 100–110, supporting the cutoffs we used to separate different conditions.

## Discussion

Using a task that simulated different win–lose situations, we found different BOLD signal responses in win and lose trials in different win–lose situations.

Lose Trials in WIN, LOSS, and TIE

### Experience of Negative Emotion

More intense activity in the bilateral insula and superior temporal gyrus was observed in LOSS than in WIN and TIE. The insula significantly influences subjective emotional experience. Functional imaging studies have revealed that the insula considerably affects the experience of several basic emotions, especially negative ones, including anger, fear, disgust, and sadness [7], [16]. The insula can also be activated even when a person merely imagines pain [17]. Moreover, a large volume of anterior insula gray matter correlates with increased accuracy in the subjective sense of negative emotional experience [18]. These findings are supported by the correlation between insula activity and the negative experience of participants in this study. The superior temporal gyrus is involved in the perception of negative emotions [19], [20]. The negative trend of activity in the superior temporal gyrus reflects a redistribution of resources from areas implicated in cognitive processing to those directly involved in emotion processing [21].

In this study, more intense activity in the insula and superior temporal gyrus was observed in lose trials in LOSS than in WIN and TIE. Participants also reported stronger negative experiences in lose trials in LOSS than in WIN. The correlation between insula activation and the negative experience suggests that intense insula activity is related to strong negative experience. Considering the behavioral and imaging results, we infer that the participants experienced stronger negative emotions in lose trials in LOSS than in WIN and TIE.

### Desire to Win

As we hypothesized, reward-related brain regions were activated in lose trials. The OFC is important in signaling the expected rewards/punishments of an action given the particular details of a situation [3], [5], [9]. Neuroimaging studies in humans have found that OFC regions are activated by pleasant touch and reward [22], [23]. The highly activated OFC observed in the present study suggests that the desire of participants to win was enhanced in LOSS conditions. The superior frontal gyrus (SFG) is an important part of the reward circuit. The SFG is a specific prefrontal cortical region that supports cue-induced craving. A study of nicotine addiction found that the SFG significantly affects the modulation of craving for attraction; highly intense activity in the SFG in response to smoking cues versus neutral cues was observed [24]. In this study, participants reported stronger craving for winning in LOSS than in WIN. Correlation results support the conclusion that SFG activation is related to the desire to win. According to the features of the task and the functions of the brain areas, the intense OFC and SFG activity in LOSS suggests that the participants showed a heightened desire to win in lose trials in LOSS than in WIN and TIE.

### Executive Control

Other robust activations are located in the cingulate cortex, including the right ACC and left posterior cingulated cortex (PCC). The ACC is an important part of the network of executive functions, including inhibitory control and conflict processing [25], [26]. In this study, the surviving ACC cluster in the rostral area is responsible for the emotional aspects of conflict monitoring [27]. The PCC serves multiple functions, including an active role in cognitive control [28], [29]. The PCC is involved in broad-information gathering, which is necessary to control responses to a rapidly changing environment [30]. In this study, more intense ACC activity was observed in lose trials in LOSS than in WIN. The beta figures showed that the difference in ACC was caused by decreased brain activity in WIN and increased brain activity in LOSS. The negative correlation between ACC/PCC activation and subjects' experience of losing in LOSS suggest that the participants who experienced strong negative emotion had intense ACC/PCC activity. This finding supports the effect of the ACC/PCC on emotion regulation during this process. Thus, intense activity in cingulate cortices suggests that the participants engaged in more cognitive activities in the executive process to regulate their emotion or impulse during lose trials in LOSS than in WIN and TIE.

### Win Trials in WIN, LOSS, and TIE

In win trials, the only difference between LOSS and TIE was observed in the PCC. No other significant difference was found among WIN, LOSS, and TIE. The posterior cingulate cortex is involved in executive control [28], [29], as discussed above. The intense brain activity in PCC in LOSS suggests that participants engaged more cognitive activities to control their experience (i.e., excitement) in LOSS than in WIN and TIE.

### Limitations

Several limitations should be considered in the current study. First, no behavioral measures were performed in the reward/punishment experience process. Second, we did not measure participants' emotional experience when they were performing the task in scanner, we only asked them to recall their experience in different situations out of scanner.

### Conclusions

In summary, the participants' experience of winning trials had great similarity among different win–lose situations. However, the participants' experience of losing trials significantly varied among different win–lose situations. The participants who experienced strong negative emotion had a strong desire to win and engaged intense cognitive activity to control their emotion/craving in lose trials in LOSS than in WIN and TIE.

## Supporting Information

Table S1 **The self-reported emotion experience questionnaire.**
(DOC)
