# Supplementary material for: Brain Activity in Advantageous and Disadvantageous Situations: Implications for Reward/Punishment Sensitivity in Different Situations
Source: PLoS One. 2013 Nov 12;8(11):e80232. doi: 10.1371/journal.pone.0080232 (PMC3827214; doi:10.1371/journal.pone.0080232)
Supplement: Table S1 — The self-reported emotion experience questionnaire. (DOC) [file pone.0080232.s001.doc]

Table S1. The self-reported emotion experience questionnaire

Please recall your experience in different situations during experiment.

| Did you notice that your account has ever more than 100 Yuan? | Yes No  If yes, please answer the follow questions |
| --- | --- |
| In this situation, when you won 10 Yuan, your experience was | Extreme extreme  negative positive  1 2 3 4 5 6 7 |
| In this situation, when you lose 10 Yuan, your experience was | Extreme extreme  negative positive 1 2 3 4 5 6 7 |
| In this situation, how strong were your craving for win | Not at all extreme strong  1 2 3 4 5 6 7 |
|  |  |
| Did you notice that your account has ever more than 100 Yuan? | Yes No  If yes, please answer the follow questions |
| In this situation, when you won 10 Yuan, your experience was | Extreme extreme  negative positive  1 2 3 4 5 6 7 |
| In this situation, when you lose 10 Yuan, your experience was | Extreme extreme  negative positive 1 2 3 4 5 6 7 |
| In this situation, how strong were your craving for win | Not at all extreme strong  1 2 3 4 5 6 7 |
